# The Impact of Antibiotic Use on Mortality in Patients Hospitalized in a COVID-19 Centre from Romania: A Retrospective Study

**DOI:** 10.3390/medicina58111628

**Published:** 2022-11-11

**Authors:** Maria-Ilinca Iosub, Elena-Sabina Balan, Larisa Pinte, Ana-Maria Draghici, Cristian Baicus, Camelia Badea

**Affiliations:** 1 Department of Internal Medicine, Colentina Clinical Hospital, 020125 Bucharest, Romania; 2 Cardiology Department, Colentina Clinical Hospital, 020125 Bucharest, Romania; 3 Faculty of Medicine, Carol Davila University of Medicine and Pharmacy, 050474 Bucharest, Romania

**Keywords:** COVID-19, antibiotic therapy, mortality, female gender, neoplasm history, heart failure, diabetes mellitus

## Abstract

*Background and Objectives*: Considering the significant number of patients worldwide that received empirical antibiotic therapy for COVID-19 infection due to their critical condition and the lack of therapeutical guidelines, we wanted to find out the consequences of antibiotic use in our study population. *Materials and Methods*: We conducted a retrospective cohort study including symptomatic patients older than 18 years, hospitalized for SARS-CoV-2 between March and December 2020 in the Internal Medicine and Pneumology Departments of Colentina Clinical Hospital. The elected outcome was death, while independent variables were antibiotic therapy and literature-cited parameters associated with mortality in this disease. *Results*: Out of 198 included patients, 96 (48.48%) patients received antibiotic therapy during hospitalization. Female gender (OR = 2.61, *p* = 0.04), history of neoplasm (OR = 7.147, *p* = 0.01), heart failure (OR = 8.62, *p* = 0.002), and diabetes mellitus (OR = 3.05, *p* = 0.02) were significantly associated with death in multivariate analysis. Antibiotic treatment showed a higher probability of death both in bivariate (OR = 5.333, *p* < 0.001) and multivariate analysis adjusted for the aforementioned prognostic factors (OR = 3.55, *p* = 0.01). *Conclusions*: After adjusting for confounders, in-hospital antibiotic administration did not improve survival in COVID-19 patients.

## 1. Introduction

The year 2020 was seriously marked by the unforeseeable evolution of the COVID-19 pandemic. The public health system worldwide was constantly suffering from a lack of means in predicting and treating this novel infection. Doctors faced the challenge of having to apply different methods of treatment when the guidelines offered no more answers. An important issue was the extensive prescription of antibiotics even when no bacterial co-infection or secondary infection was involved.

There is a big concern nowadays with multi-resistant bacterial infections that lead to around 700,000 deaths per year, and the number is estimated to reach 10 million deaths by the year 2050 [1]. Having this in mind it is impossible not to worry about the extensive use of antibiotics since the pandemic has started. A literature review about this subject reports that empirical antibiotic therapy was given to even more than 70% of the hospitalized patients for COVID-19 infection [2]. Another retrospective study from a southern European country stated that 21.6% of the hospitalized patients were prescribed no antibiotics, 43.9% were appropriately prescribed antibiotics, and 34.2% were inappropriately prescribed antibiotics [3]. Another literature review that included studies from all over the world showed that 8501 out of 10,329 COVID-19 patients (82.3%) were prescribed antibiotics [4]. An important prospective cohort study from UK pointed out that 85.2% patients with inpatient antimicrobial data received antimicrobial therapy at some point during their admission (the rate was highest for patients in critical care) [5].

On the other hand, as a response to this exponential trend in treating COVID-19 patients, new guidelines appeared concerning the use of antibiotics. WHO guidelines recommend no antibiotic therapy or prophylaxis for patients with a moderate form of COVID-19 unless signs and symptoms of a bacterial infection exist, while for severe forms of the disease, a daily assessment for de-escalation of antibiotics is required. The March 2021 UK National Institute for Health and Care Excellence (NICE) rapid guideline on managing COVID-19 pleads for not using antibiotics in the prevention or treatment of COVID-19 infection and that the only recommendation for antibiotics use is when there is a strong clinical or paraclinical suspicion of bacterial infection [6].

Taking care of hospitalized patients daily, we had witnessed a progressively worsening situation that seemed almost impossible to manage.

The main aim of our study was to unveil the effect of antibiotic therapy on mortality among the patients hospitalized in a COVID-19 centre from Bucharest, Romania and to see if our results corresponded to those published worldwide. We were also interested in finding out other parameters that influenced the prognostic of our patients.

## 2. Materials and Methods

This is a retrospective study that included symptomatic patients older than 18 years, hospitalized for COVID-19 infection between March and December 2020 at the Internal Medicine and Pneumology Departments of Colentina Clinical Hospital, Bucharest, Romania, confirmed either with a PCR or rapid antigen test. The exclusion criteria were: asymptomatic patients, patients that needed transferred to the ICU, and the ones who could not provide us an exact date for the onset of their symptoms.

Data collection was based on the observational charts of the hospitalized patients. Demographic, clinical, biological, imaging, treatment, and evolution data were analysed. Disease severity was classified as mild—symptomatic, but without dyspnea or abnormal chest imaging, moderate—if evidence of lower respiratory disease is present, but with an oxygen saturation (SpO2) above 94% on room air, or severe—patients that exhibited pulmonary infiltrates on more than 50% of the lung parenchyma accompanied by an SpO2 below 94% [7].

Comorbidities were summarized by the Charlson comorbidity index and obesity was clinician-defined. The sample was divided in two groups, one that received and one that did not receive antibiotic therapy during hospitalization. The identification of bacterial infection was based on blood, urine, sputum, and stool microbiological analysis in patients with a high clinical (presence of fever, purulent cough) and paraclinical likelihood (raised level of procalcitonin, C-reactive protein, consolidation on chest computed tomography).

The outcome was mortality. The independent variables were antibiotic prescription, and the clinical and biological parameters showed by the previous studies as associated with a higher mortality. As the ISARIC score (4C mortality score) [8] was found to be the most valid for prognosis, we used its variables for adjustment. Additionally, this model showed that ≥2 comorbidities showed an 80% increase in mortality, and we used this for adjustment too.

The statistical analysis was performed using SPSS Version 20.0. An Internet-based calculator (EBM calculator—Knowledge Translation Program, Toronto, ON, Canada) was used for calculation of relative risk with confidence interval [9].

The variables associated with death with *p* < 0.10 in bivariate analysis were evaluated in a logistic regression model. The variables were selected for logistic regression with the forward conditional method because of the relative low sample size. Hypothesis testing was 2-tailed. Statistical significance was defined by *p* < 0.05.

**Figure 1:**
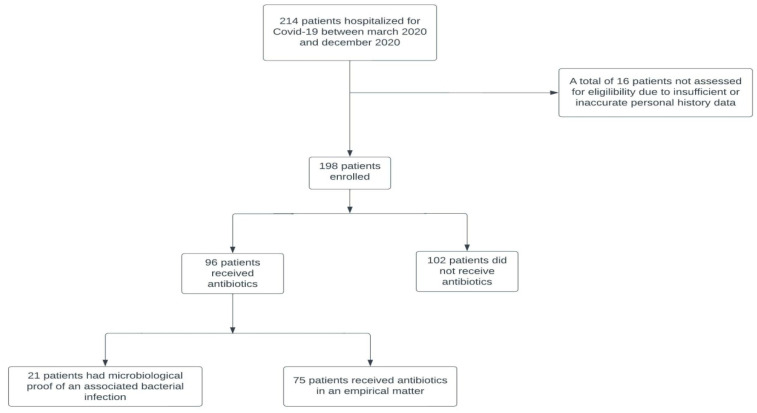
Study design.

The design of our study is summarized in the following flowchart (Figure 1). Firstly, we performed bivariate and multivariate analysis on the entire sample. Secondly, because it implies another antibiotic course, as well as increased inflammation and mortality, we excluded patients with a positive *Clostridioides difficile* test, and afterwards we also excluded any other patient with microbiological evidence of a superimposed bacterial infection. On the resulting subgroups we applied the same statistical tests as we did on the initial sample, comparing the retrieved results.

## 3. Results

Following the exclusion criteria, our sample was represented by 198 patients with a slightly male predominance (109/198). The median age was 61 (23–91).

Ten (5.1%) of the patients had a mild form of disease, 91 (45.95%) had a moderate form, while 97 (48.98%) had a severe form of COVID-19.

The overall mortality rate in our sample was 15.15–13.13% with severe disease, and 2.02 % with moderate disease.

Demographic and comorbidity data distribution were summarized in Table 1.

Forty-four (22.22%) out of the 198 patients received antibiotics at home prior to the admission, while 96 (48.48%) of the patients were first administered one or more antibiotic classes during the hospitalization. Out of the patients who were prescribed antibiotics, 2 (2.1%) developed a mild disease, 41 (42.7%) had a moderate disease and 53 (55.2%) a severe form of the disease. The prescribed antibiotic classes during hospitalization were: beta-lactams (penicillins, cephalosporins, carbapenems, beta-lactams inhibitors), glycopeptides (vancomycin), tetracyclins (doxycycline, tygecycline), oxazolidonones (linezolid), macrolides (clarithromycin, azithromycin), and fluoroquinolones (Ciprofloxacin, Levofloxacin, Moxifloxacin). Because of the heterogeneous data that resulted from simultaneous or successive antibiotic regimens, it is very difficult to determine the exact percentage of each prescribed antibiotic class. Twenty-one (10.61%) patients underwent antimicrobial therapy guided by the evidence of a bacterial infection (positive blood, urine or sputum culture or *Clostridioides difficile* stool test). The following bacteria species were isolated: *Klebsiella* spp. (4 cases), *Staphylococcus aureus* (1 case), MDR *E. coli* (2 cases), *Clostridioides difficile* (14 cases). The other 75 received antibiotics in an empirical manner (based on increasing procalcitonin levels, inflammatory biomarkers, and clinical judgement).

Upon admission, based on the Charlson comorbidity index, we were able to stratify the patients’ risk of developing a milder or more severe form of the disease and anticipate what prospects they might have had. It can be regarded as a useful marker, hence its good predictive value of mortality—AUC = 0.784, *p* < 0.001, 95% CI [0.697; 0.872].

Some inflammation biomarkers showed a significantly statistic association between themselves and mortality in the bivariate analysis (*p* < 0.005)—the maximum values of C reactive protein and D-dimer.

ROC curve analysis proves that both the maximum value of the C reactive protein and the maximum value of D-dimer are good predictors of death as shown in Table 2.

In our sample there was no association between mortality and maximum level of ferritin.

In bivariate analysis we found out that antibiotic treatment shows a higher probability of death, and respectively has an OR of 5.333 with a *p*-value below 0.001. Age (*p* < 0.001), female gender, the history of a neoplasm, heart failure, and diabetes mellitus were the other parameters associated with the outcome of death, as shown in Table 1.

Additionally, the number of comorbid conditions showed a significant statistic association with mortality (*p* = 0.009). Furthermore, we tested an ISARIC score-derived parameter, the presence of at least two comorbidities, which was as well associated with in-hospital mortality (*p* = 0.03).

Multivariate analysis showed that age, neoplasm history, heart failure, and diabetes mellitus had a significant risk of death—Table 3. When adjusted for those conditions and for disease severity, association between antibiotic therapy and mortality did not remain as statistically significant.

Since the presence of a significant inflammatory state was also known as a mortality predictor, the maximum values of C reactive protein and D-dimer were added in the logistic regression model. Furthermore, we adjusted the analysis for the patients’ preexisting conditions using a more concentrated variable, an ISARIC score derived parameter (the presence of at least two comorbidities). The results are presented below, in Table 4. 

Antibiotic therapy failed to associate with mortality after being adjusted for age, gender, comorbidities, inflammatory markers and disease severity. The latter remained the most important mortality predictor (OR 3.95, *p* = 0.008).

In order to further remove other confounding variables we excluded the patients with a confirmed *Clostridioides difficile* infection, thus resulting in a new subgroup. According to the bivariate analysis patients who were prescribed antibiotics were almost five times more likely to decease (RR = 4.905, [1.957; 12.299], *p* < 0.001). If they had at least two comorbidities they had an eleven fold greater risk of mortality (RR = 11.938, [1.666; 85.563], *p* = 0.002).

Even though in the bivariate analysis there was a statistically significant association, when taking into consideration other prognostic factors, antibiotic therapy did not show a significant association with mortality after the adjustment of the logistic regression model (Table 5).

We went further with the exclusion of the patients with any proof of secondary bacterial infection, resulting in a subgroup treated with antibiotics solely in an empirical manner, in which we searched for the association between antibiotic treatment and survival.

Bivariate analysis in patients without any proven bacterial infection showed that patients who received antibiotics had a threefold higher risk of in-hospital death (RR = 3.283, 95% CI [1.358; 7.934], *p* < 0.009). Using the same type of analysis, the presence of at least two comorbidities was associated with an increase in the risk of death by eight times (RR = 8.422, [1.167; 60.801], *p* = 0.0015).

The results obtained from the logistic regression model performed in patients without any documented bacterial infection remained similar to those obtained in the earlier stage of the statistical analysis (before the exclusion of those specific participants). As presented in Table 6, antibiotic therapy failed to associate with a significant increase in mortality. Variables with a significant impact upon mortality were: disease severity, the maximal value of D-dimer, and age.

## 4. Discussion

Some of our results come as a reiteration stated in the studies on this topic [2,3,4,5,10,11,12,13,14], which proved that an important percentage of patients hospitalized worldwide for COVID-19 infection received empirical antimicrobial therapy and that this management did not lead to an improved outcome [10,15].

Some biological markers, surrogates of the cytokine storm, have shown a significantly association with the outcome of death in bivariate analysis; furthermore, they have proven themselves as good predictors of mortality in the ROC curve analysis. In this aspect we chose to use the maximum value of C reactive protein, D-dimers and ferritin from all the available values of a patient during his or her hospital stay. Our results concerning the prognostic utility of C-reactive protein correspond to the results stated in other studies [16,17]. We concluded that D-dimer levels were also associated with a higher mortality rate, in the same way that was mentioned in other studies [18,19,20]. In our sample there was no association between mortality and maximum level of ferritin, unlike the results described in other studies [21,22]. The former can also be stated about the lack of an association between procalcitonin levels and an increase in mortality in our sample.

The ISARIC 4C mortality score was validated in several papers as an efficient prognostic score [8]. Some of its components were already used in the severity stratification of patients (oxygen saturation upon admission, the presence of pulmonary infiltrates, respiratory rate) according to the NIH protocols [23]. Other parameters associated with a significant increase in mortality in the aforementioned score (age, gender, C-reactive protein, and the presence of at least two comorbidities) were used as covariates in the logistic regression model.

As antibiotic therapy is the main cause of the *Clostridioides difficile* infection, we decided to remove the patients that had this particular secondary infection in order to predict more accurately the real effect antibiotics had upon the studied outcome. Furthermore, after removing the patients that had other proof of a microbiological infection, the lack of an association between antibiotic therapy and mortality persisted.

In bivariate analysis the mortality was increased in patients that receive antibiotic therapy because the majority of patients in our group had a moderate or severe form of disease (94.9%) and besides that they also had multiple comorbidities that led to various complications (acute kidney injury, stroke, deep vein thrombosis, acute heart failure).

This therapeutic class, prescribed as a desperate act in a life-threatening situation, did not come to rescue, therefore the survival rate did not improve. Moreover, at first glance it made us think that it was rather harmful, but after careful adjusting to the other known prognostic factors we concluded that the apparent increase in mortality was due to the fact that a significant proportion of the patients were already in an aggravated state rather than the effect of the antibiotic therapy itself.

The main limitations of this study are represented by its observational (retrospective) design, the relatively small sample of patients and the monocentric approach, even though the data were rigorously collected and there were no missing data. It should be acknowledged that the resulting conclusions could not reflect the evolution of all the SARS-CoV-2 variants in relationship with antibiotic prescribing.

An important bias could be the confounding by indication (the patients with the most severe cases were preferentially treated with antibiotic therapy), which, if strong, could not have been reversed by the adjustments for the known prognostic factors. Therefore, only a randomized controlled trial will probably give us a more certain conclusion.

We underline the fact that at the time when our patients were hospitalized, the national and international guidelines were changing in a very rapid manner and also lacked clear answers regarding antibiotic therapy.

## 5. Conclusions

After adjusting for confounders, in-hospital antibiotic administration did not improve survival in COVID-19 patients.

Thus, taking into account the fact that the unnecessary usage of antibiotics does not come without consequences, the data available in the literature so far, including our study, does not support unjustified antimicrobial agent administration in COVID-19 infection.

## Figures and Tables

**Table 1:** Medical history data and its impact on mortality.

	Yes	Deceased	Alive	Relative Risk of Death	95% CI	*p*
Antibiotic	96 (48.48%)	24 (25%)	72 (75%)	5.333	[2.072; 13.726]	<0.001
No antibiotic	-	6 (5.88%)	96 (94.11%)	-	-	-
Female gender	89 (44.94%)	19 (21.34%)	70 (78.65%)	2.115	[1.063; 4.208]	0.046
Hypertension	103 (52.02%)	17 (16.51%)	86 (83.49%)	1.206	[0.62; 2.348]	0.723
Dyslipidaemia	24 (12.12%)	4 (16.66%)	20 (83.33%)	1.115	[0.426; 2.92]	0.934
Diabetes mellitus	45 (22.72%)	12 (26.66%)	33 (73.33%)	2.267	[1.183; 4.344]	0.027
Obesity	42 (21.21%)	9 (21.42%)	33 (78.57%)	1.592	[0.788; 3.214]	0.300
Atrial fibrillation	22 (11.11%)	8 (36.36%)	14 (63.63%)	2.909	[1.478; 5.725]	0.009
Chronic coronary syndrome	22 (11.11%)	5 (22.72%)	17 (77.27%)	1.600	[0.683; 3.75]	0.462
Heart failure	13 (6.56%)	9 (69.23%)	4 (30.76%)	6.099	[3.548; 10.484]	<0.001
Chronic hepatitis/cirrhosis	14 (7.07%)	3 (21.42%)	11 (78.57%)	1.460	[0.505; 4.223]	0.770
CKD	11 (5.55%)	2 (18.18%)	9 (81.81%)	1.214	[0.331; 4.452]	0.885
Asthma/COPD	17 (8.58%)	4 (23.52%)	13 (76.47%)	1.638	[0.648 to 4.143]	0.513
Stroke	5 (2.52%)	0(0%)	5 (100%)	0.530	[0.037; 7.683]	0.936
Neoplasm	10 (5.05%)	6 (60%)	4 (40%)	4.700	[2.506; 8.817]	<0.001
Autoimmunity	6 (3.03%)	2 (33.33%)	4 (66.66%)	2.286	[0.701; 7.455]	0.494
IBD/celiac disease	4 (2.02%)	1 (25%)	3 (75%)	1.672	[0.296; 9.436]	0.881
At least 2 comorbidities	141 (71.21%)	29 (20.56%)	112 (79.43%)	11.723	[1.636; 84.03]	0.002
Charlson Index	-	5 (0, 9)	2 (0, 9)	-	-	<0.001

**Table 2:** Biological markers proven as good predictors of death in ROC curve analysis.

	AUC	*p*	95% CI
The maximum value or CRP *	0.850	0.025	[0.723; 0.977]
The maximum value of D-dimers	0.802	<0.001	[0.723; 0.881]

* CRP = C-reactive protein, AUC = area under the curve, 95% CI = confidence interval.

**Table 3:** Antibiotic therapy adjusted for several comorbidities that were highly associated with mortality (logistic regression).

	B	*p*	OR, 95% CI
Antibiotic treatment	0.863	0.122	2.369, [0.795; 7.064]
Age	0.042	0.044	2.530, [1.001; 1087]
Neoplasm	2.006	0.014	7.437, [1.494; 37.016]
Heart failure	1.154	0.039	4.546, [1.080; 19.139]
Diabetes mellitus	1.007	0.056	2.737, [1.171; 7.988]
Female gender	0.928	0.068	2.530, [0.934; 6.855]
Disease severity	1.403	0.006	4.067, [1.506; 10.986]

**Table 4:** The association between antibiotic therapy and mortality—logistic regression model after the adjustment for other involved prognostic factors.

	B	*p*	OR, 95% CI
Antibiotic treatment	0.893	0.128	2.443, [0.773; 7.717]
Female gender	0.948	0.059	2.579, [0.965; 6.896]
Age	0.062	0.004	1.064, [1.020; 1.110]
Disease severity	1.374	0.008	3.951, [1.422; 10.981]
Maximal value of D-dimer	0.132	0.003	1.141, [1.044; 1.246]
Maximal value of C reactive protein	0.002	0.564	1.002, [0.996; 1008]
The presence of at least 2 comorbidities	−0.654	0.313	0.520, [0.146; 1.851]

**Table 5:** Multivariate analysis in patients without a proven *Clostridioides* infection—adjusted for age, gender, severity, inflammatory markers and the presence of at least 2 comorbidities.

	B	*p*	OR, 95% CI
Antibiotic treatment	0.872	0.174	2.391, [0.680; 8.411]
Female gender	1.048	0.056	2.851, [0.974; 8.344]
Age	0.058	0.020	1.059, [1.009; 1.112]
Disease severity	1.518	0.009	4.565, [1.469; 14.180]
Maximal value of D-dimer	0.178	0.001	1.195, [1.077; 1.327]
Maximal value of C-reactive protein	0.001	0.871	1.001, [0.994; 1.007]
The presence of at least 2 comorbidities	−1.473	0.093	0.229, [0.041; 1.276]

**Table 6:** Multivariate analysis concerning patients who received antibiotic therapy solely in an empirical manner—adjusted for age, gender, severity, inflammatory markers, and the presence of at least 2 comorbidities.

	B	*p*	OR, 95% CI
Antibiotic treatment	0.376	0.562	1.456, [0.409; 5.185]
Female gender	0.829	0.137	2.290, [0.767; 6.835]
Age	0.048	0.049	1.049, [1.000; 1.100]
Disease severity	1.159	0.008	4.906, [1.524; 15.789]
Maximal value of D-dimer	0.155	0.001	1.168, [1.062; 1.283]
Maximal value of C-reactive protein	0.002	0.585	1.002, [0.996;1.008]
The presence of at least 2 comorbidities	−1.119	0.171	0.327, [0.066;1.624]

## Data Availability

The data presented in this study are available on request from the corresponding author.
